# Ten Machine Learning Models for Predicting Preoperative and Postoperative Coagulopathy in Patients With Trauma: Multicenter Cohort Study

**DOI:** 10.2196/66612

**Published:** 2025-01-22

**Authors:** Xiaojuan Xiong, Hong Fu, Bo Xu, Wang Wei, Mi Zhou, Peng Hu, Yunqin Ren, Qingxiang Mao

**Affiliations:** 1 Department of Anesthesiology Daping Hospital Army Medical University Chongqing China; 2 Department of Anesthesiology Chongqing Emergency Medical Center, Chongqing University Central Hospital, School of Medicine Chongqing University Chongqing China; 3 Department of Anesthesiology General Hospital of Southern Theater Command of PLA Guangzhou China; 4 Department of Anesthesiology The PLA Rocket Force Characteristic Medical Center Beijing China; 5 School of Public Policy and Administration Chongqing University Chongqing China

**Keywords:** traumatic coagulopathy, preoperative, postoperative, machine learning models, random forest, Medical Information Mart for Intensive Care

## Abstract

**Background:**

Recent research has revealed the potential value of machine learning (ML) models in improving prognostic prediction for patients with trauma. ML can enhance predictions and identify which factors contribute the most to posttraumatic mortality. However, no studies have explored the risk factors, complications, and risk prediction of preoperative and postoperative traumatic coagulopathy (PPTIC) in patients with trauma.

**Objective:**

This study aims to help clinicians implement timely and appropriate interventions to reduce the incidence of PPTIC and related complications, thereby lowering in-hospital mortality and disability rates for patients with trauma.

**Methods:**

We analyzed data from 13,235 patients with trauma from 4 medical centers, including medical histories, laboratory results, and hospitalization complications. We developed 10 ML models in Python (Python Software Foundation) to predict PPTIC based on preoperative indicators. Data from 10,023 Medical Information Mart for Intensive Care patients were divided into training (70%) and test (30%) sets, with 3212 patients from 3 other centers used for external validation. Model performance was assessed with 5-fold cross-validation, bootstrapping, Brier score, and Shapley additive explanation values.

**Results:**

Univariate logistic regression identified PPTIC risk factors as (1) prolonged activated partial thromboplastin time, prothrombin time, and international normalized ratio; (2) decreased levels of hemoglobin, hematocrit, red blood cells, calcium, and sodium; (3) lower admission diastolic blood pressure; (4) elevated alanine aminotransferase and aspartate aminotransferase levels; (5) admission heart rate; and (6) emergency surgery and perioperative transfusion. Multivariate logistic regression revealed that patients with PPTIC faced significantly higher risks of sepsis (1.75-fold), heart failure (1.5-fold), delirium (3.08-fold), abnormal coagulation (3.57-fold), tracheostomy (2.76-fold), mortality (2.19-fold), and urinary tract infection (1.95-fold), along with longer hospital and intensive care unit stays. Random forest was the most effective ML model for predicting PPTIC, achieving an area under the receiver operating characteristic of 0.91, an area under the precision-recall curve of 0.89, accuracy of 0.84, sensitivity of 0.80, specificity of 0.88, precision of 0.88, *F*_1_-score of 0.84, and Brier score of 0.13 in external validation.

**Conclusions:**

Key PPTIC risk factors include (1) prolonged activated partial thromboplastin time, prothrombin time, and international normalized ratio; (2) low levels of hemoglobin, hematocrit, red blood cells, calcium, and sodium; (3) low diastolic blood pressure; (4) elevated alanine aminotransferase and aspartate aminotransferase levels; (5) admission heart rate; and (6) the need for emergency surgery and transfusion. PPTIC is associated with severe complications and extended hospital stays. Among the ML models, the random forest model was the most effective predictor.

**Trial Registration:**

Chinese Clinical Trial Registry ChiCTR2300078097; https://www.chictr.org.cn/showproj.html?proj=211051

## Introduction

Severe trauma is a significant global public health burden and a leading cause of reduced life expectancy. The Global Burden of Diseases, Injury, and Risk Factors Study 2017 estimates that trauma accounts for 8% of total deaths annually [1]. Traumatic coagulopathy (TIC) is a systemic inflammatory state accompanied by coagulation dysfunction, acidosis, and hypothermia that occurs after traumatic injury [2]. TIC is observed in one-quarter to one-third of patients with trauma [3,4] and is associated with increased rates of massive transfusion and multiple organ failure (MOF), prolonged intensive care unit (ICU) stay and length of stay (LOS), and a 4-fold increase in mortality [3]. Harhangi et al [5] reported that TIC is linked to a 9-fold increase in mortality and a 36-fold increase in the likelihood of adverse outcomes [5]. Early endogenous TIC is associated with increased bleeding, higher red blood cell (RBC) transfusion rates, and a greater risk of secondary complications like multiple organ dysfunction syndrome (MODS) and thromboembolism [6]. Cohen and Christie [7] also reported that patients with TIC have worse prognoses, including higher rates of infection, thromboembolism, acute lung injury, MOF, and mortality. Without proper and timely diagnosis and treatment, posttraumatic bleeding and associated TIC remain potentially preventable causes of MOF [8]. Therefore, the latest European guidelines indicate that the immediate identification and management of TIC can improve outcomes for severely injured patients [9].

In recent years, artificial intelligence (AI) has received significant attention for its potential use in various aspects of human activities, including health care [10]. Sidey-Gibbons and Sidey-Gibbons [11] and Liu and Salinas [12] suggested that machine learning (ML) excels over traditional methods in handling large, unstructured, nonlinear, or incomplete datasets, improving outcome predictions for patients with trauma. With the advent of ML and improved computational analysis methods, recent approaches have aimed to enhance predictions and identify which factors are most important in contributing to posttraumatic mortality [13]. Rashidi et al [14] emphasized the critical importance of rapid diagnosis and intervention for managing hemostatic disorders, which can lead to life-threatening bleeding or clotting. AI or ML models can significantly enhance turnaround times, accuracy, and the timeliness of diagnoses and interventions, potentially improving patient care and outcomes.

Our data show that despite surgical interventions such as hemostasis, vascular reconstruction, fracture reduction, organ repair, blood transfusion, and the correction of electrolyte imbalances in patients who experienced preoperative TIC, 50% of these patients still had TIC postoperatively [15]. These patients were more prone to develop complications during hospitalization, and preoperative and postoperative traumatic coagulopathy (PPTIC) was identified as an independent risk factor for these complications. Therefore, we plan to explore the risk factors for PPTIC in patients with trauma and its relationship with in-hospital complications. We will use admission indicators from patients with trauma to predict the risk of PPTIC, applying 10 different ML models to estimate the probability of PPTIC occurrence and conducting a comprehensive evaluation of each model. This predictive approach can aid clinicians in implementing timely and appropriate interventions to reduce the incidence of PPTIC and related complications, thereby lowering in-hospital mortality and disability rates for these patients.

## Methods

### Ethical Considerations

Data from 4 medical centers were anonymized and integrated. Ethical approvals were granted by the respective ethics committees: Daping Hospital, Medical Research Ethics (2023) 261; Chongqing Emergency Medical Center, 2023 ethical review (48); the People’s Liberation Army Rocket Force Characteristic Medical Center (KY 2023037); and the General Hospital of Southern Theatre Command of People’s Liberation Army (NZLLKZ2024021). As this was a retrospective study, patient informed consent was waived by the ethics committees. It was registered with the Chinese Clinical Trial Registry (ChiCTR2300078097) and adhered to the Declaration of Helsinki. This study adheres to 2 key guidelines to ensure rigorous reporting: the TRIPOD-AI (Transparent Reporting of a Multivariable Prediction Model for Individual Prognosis or Diagnosis—Artificial Intelligence) guidelines for the development and reporting of multivariable ML predictive models in biomedical research (Multimedia Appendix 1) [16] and the STROCSS (Strengthening the Reporting of Cohort Studies in Surgery) criteria, which standardize the reporting of surgical observational studies [17].

### Inclusion and Exclusion Criteria

The inclusion and exclusion criteria are as follows:

- Inclusion criteria: Patients who underwent surgical treatment after trauma from January 2017 to February 2023.
- Exclusion criteria: Patients without trauma (snake bites, bee stings, and burns); patients who did not undergo surgery or stopped treatment; patients younger than 18 years of age; patients with hematological diseases; patients with a history of liver cirrhosis; pregnant women; and patients without coagulation-related tests results either preoperatively or postoperatively.

### Data Collection

We retrieved patients who underwent trauma or concurrent surgeries using the MIMIC IV (Medical Information Mart for Intensive Care) 2.2 database, with the detailed data extraction process outlined in Multimedia Appendix 2. We then collected the necessary indicators using subject_id and hadm_id, which included the following information: ID number; sex; age; height; weight; type of surgery (elective or emergency); and preoperative comorbidities such as high blood pressure, diabetes, cerebrovascular diseases, chronic obstructive pulmonary disease, chronic bronchitis, asthma, and chronic renal insufficiency. Preoperative indicators included systolic blood pressure, heart rate (HR), saturation of pulse oximetry, and the presence of shock upon admission. The laboratory examination indicators (latest results prior to admission and first results postoperatively) included the following: complete blood count (white blood cell, lymphocyte [%], monocyte [%], neutrophil [%], eosinophil [%], basophil [%], RBC, mean corpuscular hemoglobin concentration, mean corpuscular volume, red cell distribution width, hemoglobin, hematocrit, platelet count [PLT], mean platelet volume, renal function [urea, creatinine, and blood uric acid]), liver function (albumin, alkaline phosphatase, lactate dehydrogenase, gamma-glutamyl transferase, total protein, globulin, direct bilirubin, indirect bilirubin, total bilirubin, alanine aminotransferase [ALT], and aspartate aminotransferase [AST]), electrolytes (sodium, potassium, and calcium), and coagulation function (activated partial thromboplastin time [APTT], prothrombin time [PT], international normalized ratio [INR], and D-dimer). The relevant intraoperative indicators included the need for perioperative blood transfusion. Complications during hospitalization included pulmonary embolism, sepsis, septicemia, heart failure, delirium, pulmonary edema, abnormal coagulation, endotracheal tube intubation, invasive mechanical ventilation, tracheostomy, end-stage renal disease, systemic inflammatory response, acute respiratory distress syndrome, pleural effusion, cardiac arrest, anemia, venous thrombosis, renal failure, acute kidney failure, urinary tract infection, respiratory failure, pneumonia, LOS, in-hospital death, and length of ICU stay. Data from the other 3 medical centers were collected via electronic medical records, consistent with the previously mentioned information. Our definition criteria for TIC were as follows: PLT<100×10^^9^/L; INR>1.25; PT>14 second; and APTT>36 second [18]. PPTIC was defined as patients who experienced TIC both within 24 hours before surgery (preoperative) and within 24 hours after surgery (postoperative), meeting one or more of the above TIC diagnostic criteria in both timeframes.

### Process of ML Modeling

#### Handling Missing Data

We processed the data using Python (version 3.9; Python Software Foundation), discarding variables with over 40% missing data (Multimedia Appendix 3). For binary and categorical variables, we applied 1-hot encoding. Continuous variables were imputed using k-nearest neighbor (KNN) with *k*=5, while categorical variables underwent multiple imputations with 5 iterations.

#### Data Normalization and Feature Selection

##### Data Normalization

We created a *StandardScaler* object to standardize the data. Both the training and test data were standardized via the same procedure.

##### Outlier Handling

We calculated *z* scores for the training data, considering values above 3 as outliers, and replaced these with the mean using *np.where*.

##### Feature Selection

We assessed continuous variables with the Pearson correlation coefficient and converted categorical variables to numerical ones via label encoding. Then, the Pearson correlation coefficient was calculated for each feature relative to the target variable, and the top 50 features with the highest correlations were selected. These selected features were then refined using least absolute shrinkage and selection operator regression, resulting in a final set of 25 features.

#### Dataset Splitting

**Figure 1:**
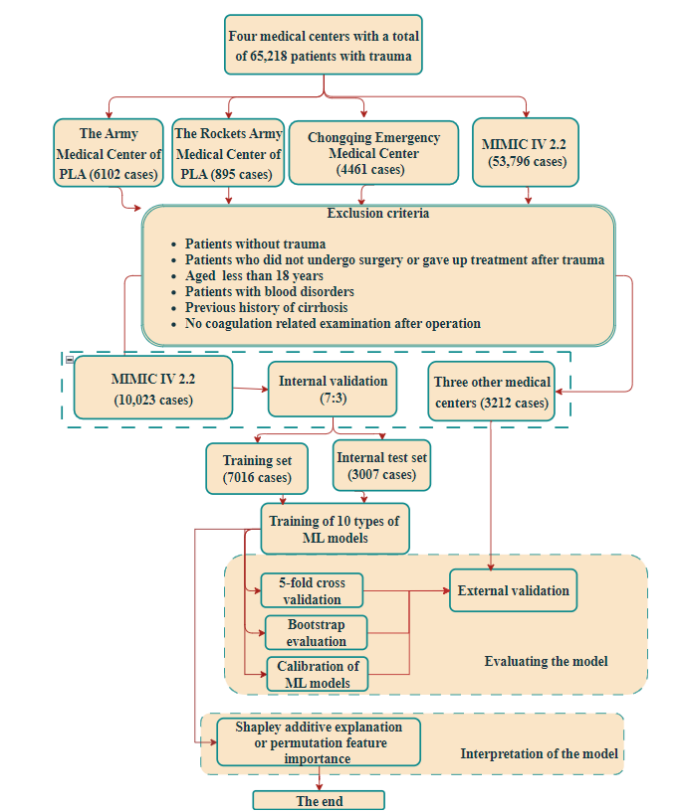
The data processing. MIMIC: Medical Information Mart for Intensive Care; ML: machine learning; PLA: People’s Liberation Army.

We modeled data from 10,023 patients with trauma in MIMIC IV, splitting it into a training set of 7631 cases and a test set of 1908 cases (7:3 ratio) using the *train_test_split* function. Additionally, we used data from 3212 patients from 3 other medical centers for external validation (Figure 1).

#### Building the ML Models

We used Python 3.9 to build the ML models, using a total of 10 models (Multimedia Appendix 4): logistic regression (LR), random forest (RF), support vector machine (SVM), decision tree (DT), KNN, gradient boosting (GB), neural networks (NN), naive Bayes (NB), AdaBoost, and extreme gradient boosting (XGBoost). We assessed the models’ performances via 7 metrics: area under the receiver operating characteristic curve (AUROC), area under the precision-recall curve (AUPRC), accuracy, sensitivity, specificity, precision, and *F*_1_-score. The Python 3.9 packages used for data processing and analysis are shown in Multimedia Appendix 5. Additionally, the full code and data processing steps, along with detailed instructions, have been made publicly available via GitHub [19].

#### Model Performance and Evaluation

In this study, we used “grid search” for hyperparameter optimization. We conducted 5-fold cross-validation by dividing the dataset into 5 subsets, using each subset once for testing and the remaining four for training. This process, repeated 5 times, helps reduce overfitting, stabilize performance evaluation, and address sample selection bias [20]. We measured accuracy, precision, recall, and *F*_1_-score.

To evaluate the robustness and generalization of 10 ML models, we used bootstrap sampling with 1000 iterations. Each iteration involved retraining the models on a bootstrap sample and calculating accuracy, precision, recall, and *F*_1_-score. We then estimated the distributions and 95% CIs for these metrics, producing 12 key indicators: accuracy mean, accuracy lower or upper 95% CI, precision mean, precision lower or upper 95% CI, recall mean, recall lower or upper 95% CI, *F*_1_-score mean, and *F*_1_-score lower or upper 95% CI.

To assess model calibration, we used the Brier score (BS), which ranges from 0 to 1, with lower scores indicating better accuracy of predicted probabilities. We trained the model, evaluated predictions on the test set, computed the BS, and plotted a calibration curve to visualize the model’s performance.

#### Interpretation of the ML

We use Shapley additive explanation (SHAP) values to assess the significance of variables in the model by evaluating their impact on predictions across all feature subsets and averaging these effects [21]. We used the Python package *SHAP* to visualize and rank the top 15 variables with the highest SHA*P* values related to PPTIC. These variables were integrated into the model, and SHA*P* values ranging from 0 to 1 were used for risk interpretation. We also conducted univariate logistic regression on these top 15 variables.

### Statistics

Statistical analysis was performed using SPSS Statistics (version 27.0; IBM Corp). Patients were divided into PPTIC and non-PPTIC groups, with PPTIC as the independent variable and other factors as dependent variables. Categorical data are presented as percentages, and continuous data as mean (SD). Depending on the distribution, continuous variables were assessed via 2-tailed *t* tests or Mann-Whitney *U* tests, and categorical variables via chi-square tests or exact tests. Univariate analysis identified risk factors for persistent trauma-induced coagulopathy, and multivariable logistic regression analyzed complications during hospitalization in the PPTIC group. A *P* value less than .05 was considered statistically significant.

## Results

### Basic Information of the Patients

We ultimately included 13,237 patients with trauma, among whom 6586 (49.8%) experienced PPTIC. The mean age of our patients was 62.45 (SD 17.85) years. In the PPTIC group, the average age was 63.6 (SD 17.4) years, whereas in the non-PPTIC group, it was 61.3 (SD 18.2) years, with *P*<.001 (Table 1). Among the patients, there were 5590 (42.2%) male individuals and 7646 (57.8%) female individuals, and this difference was significant (*P*<.001). The racial distribution included 3452 (26.1%) Asian individuals, 7402 (55.9%) White individuals, 342 (2.6%) Black individuals, 940 (7.1%) Latino individuals, and 1101(8.3%) individuals of other races.

**Table 1:** The basic information of the patients.

	PPTIC^a^ group	Non-PPTIC group	*P* value^b^
Age (years), mean (SD)	63.6 (17.4)	61.3 (18.2)	<.001
Weight (kg), mean (SD)	74.9 (23.8)	71.8 (20)	.002
BMI (kg/m^2^), mean (SD)	27.9 (8)	27.3 (7.4)	.001
Admission HR^c^, mean (SD)	86.3 (19.3)	84.6 (17)	<.001
Admission SBP^d^ (mmHg), mean (SD)	125 (22.1)	131.3 (131.4)	.001
Pre-hematocrit (%), mean (SD)	31.9 (6.4)	34.5 (6.5)	<.001
Pre-hemoglobin (g/L), mean (SD)	104.1 (22)	113.7 (22.6)	<.001
Pre-RBC^e^ (×10×12/L), mean (SD)	3.5 (0.8)	3.8 (0.7)	<.001
Pre-ALT^f^ (U/L), mean (SD)	69.6 (258.2)	44.4 (86)	<.001
Pre-AST^g^ (U/L), mean (SD)	87.2 (305.3)	48.4 (101.2)	<.001
Pre-GGT^h^ (U/L), mean (SD)	54.1 (104)	46.4 (95.7)	.031
Pre-calcium (mmol/L), mean (SD)	2.1 (0.2)	2.2 (0.2)	<.001
Pre-sodium (mmol/L) mean (SD)	0.0552	0.0476	<.001
Pre-PT^i^ (s), mean (SD)	18.3 (7.9)	12.8 (1.8)	<.001
Pre-APTT^j^ (s), mean (SD)	40.3 (13.5)	30.8 (6.7)	<.001
Pre-PLT^k^ (×10×9/L), mean (SD)	212.5 (124.3)	232.8 (103)	<.001
Pre-INR^l^, mean (SD)	1.6 (0.7)	1.1 (0.2)	<.001
Post-INR, mean (SD)	1.6 (0.6)	1.1 (0.1)	<.001
Post-PT (seconds), mean (SD)	17.5 (6.7)	12.9 (1.5)	<.001

^a^PPTIC: preoperative and postoperative traumatic coagulopathy.

^b^*P* value less than .05 was considered statistically significant.

^c^HR: heart rate.

^d^SBP: systolic pressure.

^e^RBC: red blood cell

^f^ALT: alanine aminotransferase.

^g^AST: aspartate amino transferase.

^h^GGT: glutamyl transferase

^i^PT: prothrombin time

^j^APTT: activated partial thromboplastin time.

^k^ALT: alanine aminotransferase.

^l^INR: international normalized ratio.

### Results of the ML Models

#### Training Results of the ML Models

**Figure 2:**
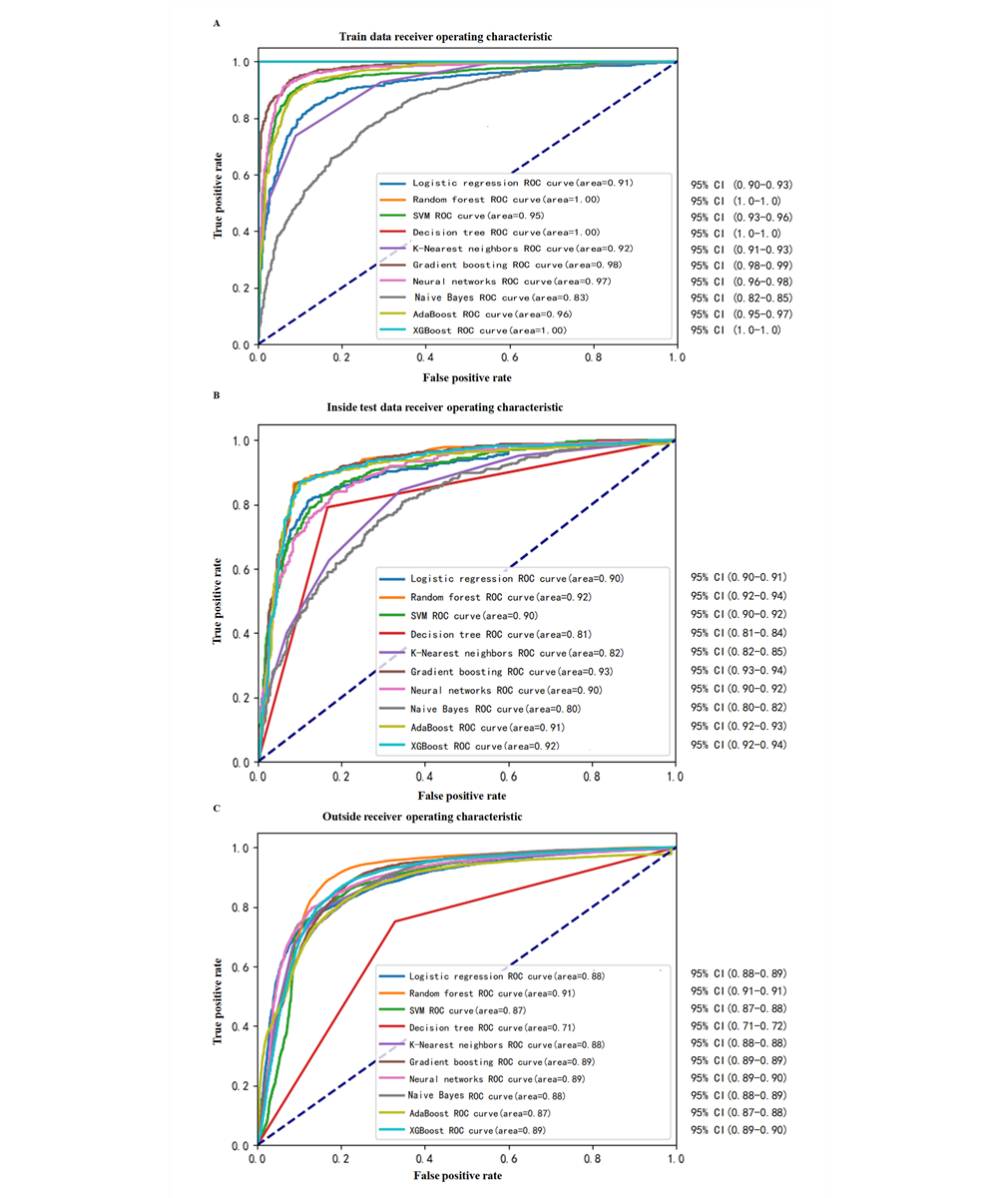
The AUROC curves of 10 machine learning models in the (A) training, (B) test, and (C) external validation set for predicting PPTIC in patients with trauma. The ROC curve for predicting PPTIC in patients with trauma is defined by the false positive rate on the x-axis, representing the proportion of negative cases misclassified as positive, and the true positive rate on the y-axis, reflecting the proportion of correctly identified positive cases (sensitivity), with both axes ranging from 0 to 1. The AUROC is a key metric for assessing the model’s predictive performance, where a higher AUROC signifies superior discriminatory ability in predicting PPTIC and improved generalizability. AUROC: area under the receiver operating characteristic curve; PPTIC: preoperative and postoperative traumatic coagulopathy; ROC: receiver operating characteristic curve; SVM: support vector machine.

We developed 10 ML risk models for predicting PPTIC. In the training set, all 10 models achieved an AUROC of 0.83 or higher, with RF, DT, and XGBoost reaching AUROC values of 1. In the internal testing set, RF achieved an excellent AUROC of 0.92, whereas the AUROC of SVM was 0.9, that of GB was 0.93, that of NN was 0.9, that of AdaBoost was 0.91, and XGBoost performed well (Figure 2). RF, GB, and XGBoost demonstrated superior performance in both the training and testing sets, with AUROC, accuracy, sensitivity, specificity, precision, and *F*_1_-score all above 0.8 (Multimedia Appendix 6).

#### Evaluating the ML Models

##### Results of the 5-Fold Cross-Validation

The results from the 5-fold cross-validation (Multimedia Appendix 7) showed that our RF, GB, AdaBoost, and XGBoost models consistently performed well across diverse subsets, demonstrating high accuracy and consistency. The SDs of the accuracy, precision, recall, and *F*_1_-scores were minimal, confirming the robustness and reliability of these ML models.

##### Bootstrap Results

The bootstrap analysis results indicated that RF, DT, GB, AdaBoost, and XGBoost achieved average accuracy, precision, recall, and *F*_1_-score all exceeding 0.91, accompanied by narrow 95% CIs (Table 2). This suggests consistently high accuracy across various sample resampling conditions, underscoring the robustness of these models. Moreover, RF, DT, GB, AdaBoost, and XGBoost demonstrated substantial stability and reliability across their respective performance metrics.

**Table 2:** The bootstrap results of 10 kinds of machine learning models.

	Mean accuracy	95% CI accuracy	Mean precision	95% CI precision	Mean recall	95% CI recall	Mean *F*_1_-score	95% CI *F*_1_-score
LR^a^	0.766	0.713-0.821	0.766	0.712-0.821	0.766	0.713-0.821	0.763	0.709-0.82
*RF^b,c^*	*0.961*	*0.956-0.966*	*0.961*	*0.956-0.966*	*0.961*	*0.956-0.966*	*0.961*	*0.956-0.966*
SVM^d^	0.573	0.572-0.573	0.503	0.328-0.755	0.573	0.572-0.573	0.419	0.417-0.421
DT^e^	0.934	0.926-0.941	0.935	0.926-0.941	0.934	0.926-0.941	0.934	0.926-0.941
KNN^f^	0.661	0.651-0.673	0.657	0.647-0.669	0.661	0.651-0.673	0.656	0.645-0.668
*GB^g^*	*0.918*	*0.914-0.923*	*0.918*	*0.914-0.923*	*0.918*	*0.914-0.923*	*0.918*	*0.914-0.923*
NN^h^	0.633	0.49-0.742	0.697	0.625-0.746	0.633	0.49-0.742	0.594	0.399-0.743
NB^i^	0.728	0.689-0.75	0.74	0.713-0.759	0.728	0.689-0.75	0.724	0.685-0.749
AdaBoost	0.892	0.885-0.899	0.892	0.885-0.898	0.892	0.885-0.899	0.891	0.884-0.898
*XGBoost^j^*	*0.96*	*0.954-0.964*	*0.96*	*0.954-0.964*	*0.96*	*0.954-0.964*	*0.96*	*0.954-0.964*

^a^LR: logistic regression.

^b^RF: random forest.

^c^Italics are used to highlight the better-performing machine learning models in our study.

^d^SVM: support vector machine

^e^DT: decision tree.

^f^KNN: k-nearest neighbor.

^g^GB: gradient boosting.

^h^NN: neural networks.

^i^NB: naive Bayes.

^j^XGBoost: extreme gradient boosting.

##### Calibration and Precision-Recall of the ML Models

**Figure 3:**
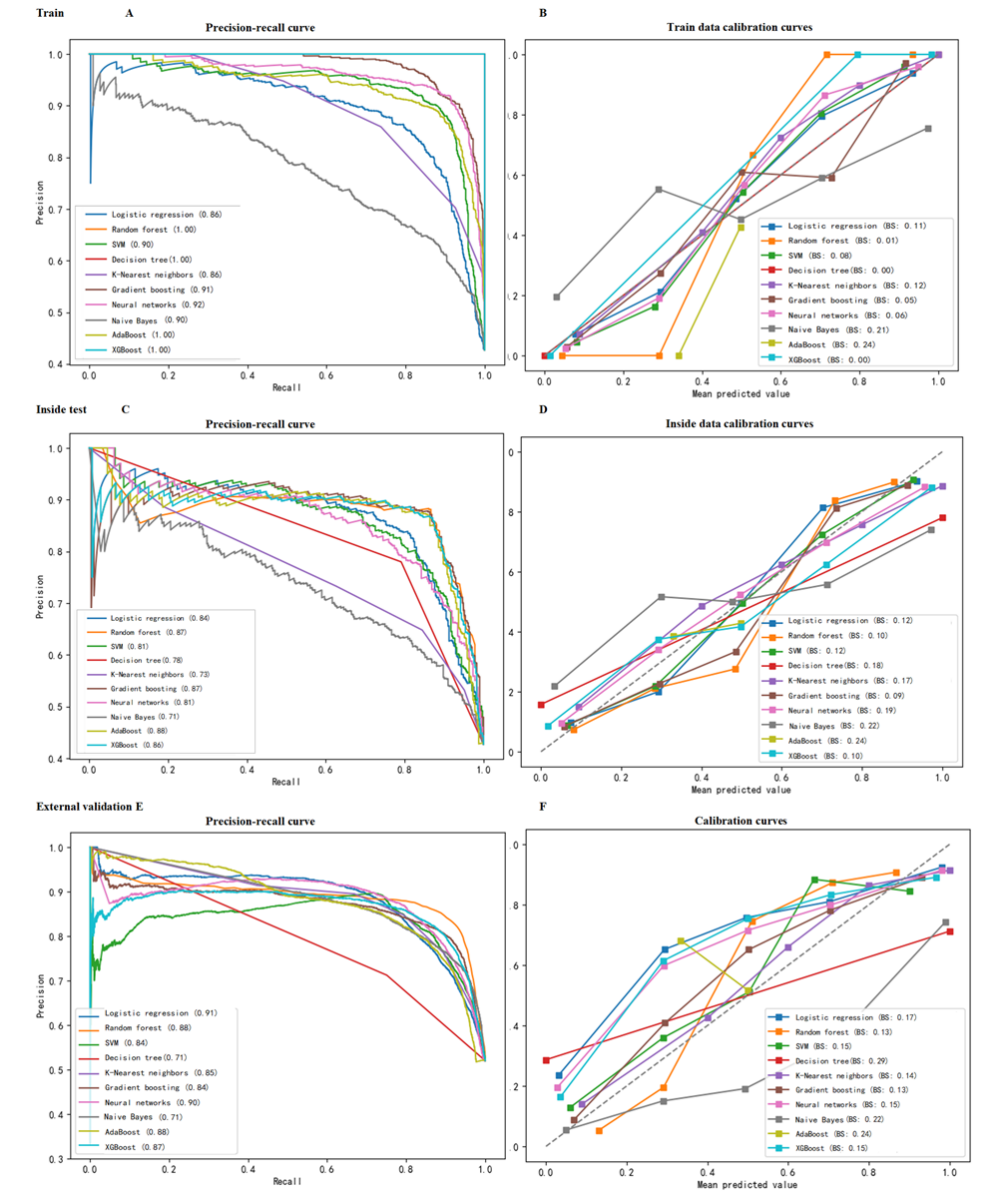
The precision-recall curve and train data calibration curve of 10 machine learning models in (A-B) train, (C-D) internal test, and (E-F) external validation sets predicted patients with PPTIC. PPTIC: preoperative and postoperative traumatic coagulopathy; SVM: support vector machine.

In the training set, RF, DT, GB, and XGBoost achieved BSs of 0.01, 0.00, 0.05, and 0.00, respectively, with precision-recall values above 0.9, demonstrating robustness. In the internal testing set, RF, GB, and XGBoost had BSs of 0.1, 0.09, and 0.10, and precision-recall values of 0.87, 0.87, and 0.86. During external validation, they recorded BSs of 0.13, 0.13, and 0.15, with precision-recall values around 0.88, 0.84, and 0.87. The BS and precision-recall values of RF, GB, and XGBoost in the external validation set closely mirrored those in the internal testing set (Figure 3), underscoring the consistency and reliability of these models.

##### Results of the External Validation of the ML Models

In the external validation set, RF, GB, NN, and XGBoost achieved AUROC values of 0.91, 0.89, 0.89, and 0.89, respectively (Figure 2), while other models attained approximately 0.87. RF showed the highest performance with AUROC 0.91 (95% CI 0.90-0.913), AUPRC 0.89 (95% CI 0.88-0.90), accuracy 0.84 (95% CI 0.83-0.85), sensitivity 0.80 (95% CI 0.79-0.81), specificity 0.88 (95% CI 0.87-0.89), precision 0.88 (95% CI 0.87-0.89), and *F*_1_-score 0.84 (95% CI 0.83-0.84). Despite being slightly lower than in the training set, RF’s performance remained stable, demonstrating strong generalizability. Thus, RF was identified as the optimal model based on 5-fold cross-validation, bootstrap, calibration, precision-recall, and external validation results.

### Interpretation of the ML Models

**Figure 4:**
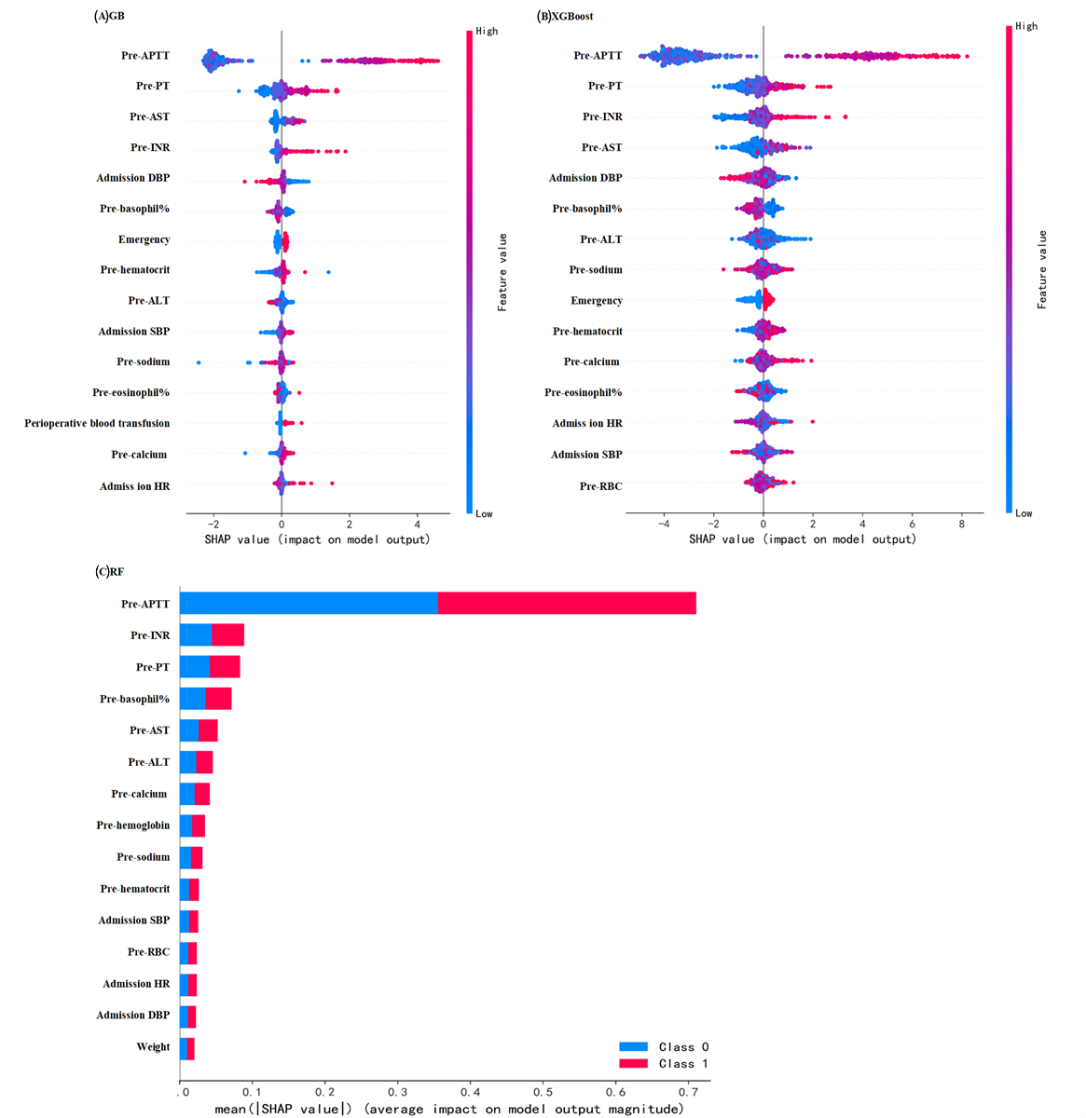
SHA*P* values of (A) GB, (B) XGBoost, and (C) RF for predicting the risk of TIC before and after surgery in patients with trauma. We selected the three best-performing ML models (GB, XGBoost, and RF) from a set of 10 to predict PPTIC outcomes and evaluated their SHA*P* values. The SHAP plot highlights the top 15 features with the most significant impact on model predictions, ranked in descending order of importance from top to bottom. The x-axis represents the SHA*P* value, while the y-axis lists the corresponding feature names. Red points represent higher feature values, while blue points represent lower feature values. If red points are skewed toward the positive direction (higher SHA*P* values), it indicates that higher feature values contribute positively to the prediction outcome (increasing the predicted value). Conversely, if blue points are skewed toward the negative direction (lower SHA*P* values), it suggests that lower feature values contribute negatively to the prediction outcome (decreasing the predicted value). ALT: alanine aminotransferase; APTT: activated partial thromboplastin time; AST: aspartate aminotransferase; DBP: diastolic blood pressure; GB: gradient boosting; HR: heart rate; PPTIC: preoperative and postoperative traumatic coagulopathy; PT: prothrombin time; RBC: red blood cell; RF: random forest; SBP: systolic pressure; SHAP: Shapley additive explanation; TIC: traumatic coagulopathy.

Analysis of SHA*P* values highlighted the key features influencing model predictions. For RF, GB, and XGBoost (Figure 4), as well as the other 7 ML models (Multimedia Appendix 8), we observed the top 15 variables ranked by their average impact. In RF, the primary predictors of PPTIC in patients with trauma were preoperative APTT, PT, INR, AST, ALT, calcium, hemoglobin, sodium, hematocrit, admission SBP, RBC, admission HR, and the need for emergency surgery or perioperative blood transfusion (Table 3). Univariate logistic regression identified prolonged APTT, PT, and INR; low levels of hemoglobin, hematocrit, RBC, calcium, and sodium; elevated ALT and AST; and the need for emergency surgery or blood transfusion were significant risk factors for PPTIC.

**Table 3:** Univariate logistic regression of risk factors for preoperative and postoperative TIC^a^ in patients with trauma.

	OR^b^ (95% CI)	*P* value^c^
Pre-APTT^d^ (s)	1.14 (1.13-1.14)	<.001
Pre-PT^e^ (s)	1.99 (1.92-2.05)	<.001
Pre-INR^f^	1289.15 (936.81-1773.99)	<.001
Pre-hemoglobin (g/L)	0.98 (0.98-0.98)	<.001
Pre-hematocrit (%)	0.94 (0.94-0.95)	<.001
Pre-RBC^g^ (×10×12/L)	0.61 (0.57-0.64)	<.001
Pre-calcium (mmol/L)	0.4 (0.33-0.49)	<.001
Pre-sodium (mmol/L)	0.96 (0.95-0.97)	<.001
Admission SBP^h^ (mmHg)	0.99 (0.99-0.99)	<.001
Admission HR^i^	1.004 (1.004-1.006)	.005
Emergency	1.34 (1.24-1.45)	<.001
Pre-ALT^j^ (U/L)	1.001 (1.001-1.002)	<.001
Pre-AST^k^ (U/L)	1.001 (1.001-1.002)	<.001
Perioperative blood transfusion	1.42 (1.12-1.81)	.004

^a^TIC: traumatic coagulopathy.

^b^OR: odds ratio.

^c^*P* value less than .05 was considered statistically significant.

^d^APTT: activated partial thromboplastin time.

^e^PT: prothrombin time.

^f^INR: international normalized ratio.

^g^RBC: red blood cell.

^h^SBP: systolic pressure.

^i^HR: heart rate.

^j^ALT: alanine aminotransferase.

^k^AST: aspartate amino transferase.

### Complications During Hospitalization in Patients With PPTIC

**Figure 5:**
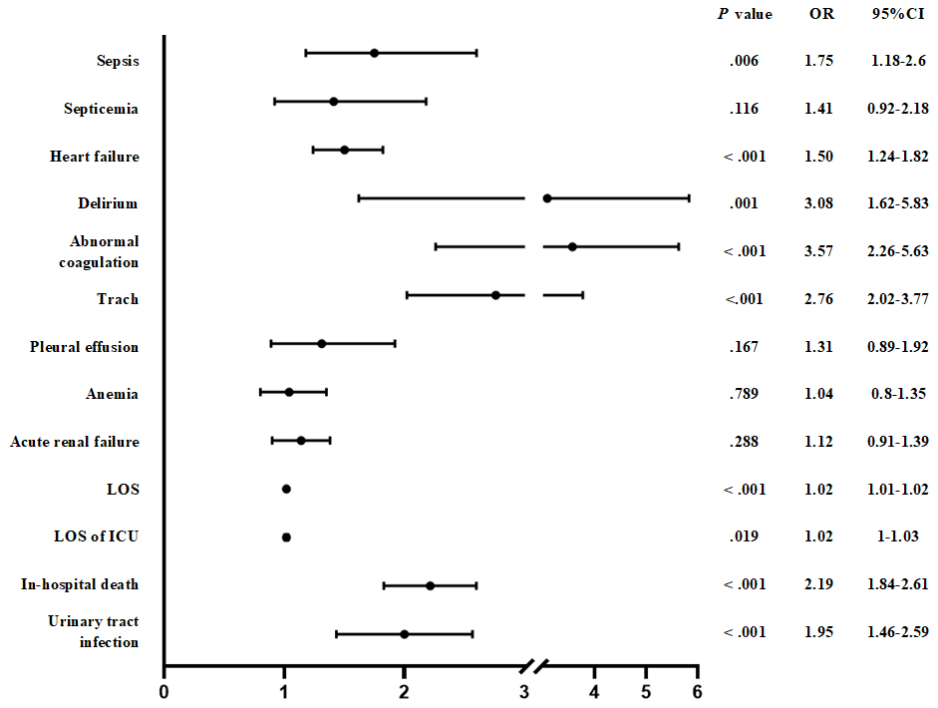
Multivariate logistic regression of postoperative complications in patients with trauma who developed TIC both before and after surgery. LOS: length of stay; ICU: intensive care unit; OR: odds ratio; TIC: traumatic coagulopathy. *P* value˂.05 was considered statistically significant.

Multivariate logistic regression analysis showed that compared with patients with non-PPTIC, those with PPTIC had significantly higher postoperative risks of the following complications: sepsis (1.75-fold, 95% CI 1.18-2.6; *P*=.006), heart failure (1.5-fold, 95% CI 1.24-1.82; *P*<.001), delirium (3.08-fold, 95% CI 1.62-5.83; *P*=.001), abnormal coagulation (3.57-fold, 95% CI 2.26-5.63; *P*<.001), tracheostomy (2.76-fold, 95% CI 2.02-3.77, *P*=.001), in-hospital mortality (2.19-fold, 95% CI 1.84-2.61; *P*<.001), urinary tract infection (1.95-fold, 95% CI 1.46-2.59; *P*<.001), and longer LOS and ICU stay (Figure 5).

## Discussion

### ML Models for Predicting PPTIC in Patients With Trauma

Our study found that RF, GB, and XGBoost had AUROC values above 0.89 for predicting PPTIC, with RF performing best at 0.91. RF showed excellent performance across training, testing, and external validation sets. RF is an ML algorithm capable of capturing potential nonlinear relationships between risk factors and outcomes [22]. It operates by creating hundreds or thousands of smaller decision trees and combining their outputs for prediction [23]. This approach helps overcome the tendency of decision trees to overfit [23] while enhancing model generalizability [24]. Therefore, we recommend the use of the RF to predict the occurrence of PPTIC in patients with trauma. The prediction variables were primarily continuous laboratory indicators, eliminating the need for conversion into binary or categorical forms and facilitating practical use. Physicians can directly input these indicators into the RF model to assess the risk of PPTIC in patients with trauma. Recent guidelines highlight the importance of early detection and management of TIC to improve outcomes for severely injured patients [9]. Our study aims to predict PPTIC using preoperative indicators to facilitate early intervention and thereby improve clinical outcomes in such patients.

Most robust PPTIC prediction models in our study are tree-based, such as RF, DT, GB, and XGBoost, a trend also seen in other coagulopathy-related ML models. Perkins et al [25] used BN to predict early TIC, defined as an INR>1.2. Their model, trained on 600 patients with trauma, achieved an AUROC of 0.93 with a BS of 0.06 in the development dataset and an AUROC of 0.95 with a BS of 0.05 in the validation set [25]. In comparison, Our RF model achieved an AUROC of 1 in training, 0.92 in validation, and 0.91 in external validation, with BS values of 0.01, 0.10, and 0.13, respectively. Li et al [26] defined acute traumatic coagulopathy (ATC) as an INR>1.5 and collected data from 818 patients with trauma admitted to the emergency department. They used LR and RF to predict ATC occurrence, with LR achieving an AUROC of 0.858 and RF achieving an AUROC of 0.830 in the internal validation set [27]. Their study used only 2 ML models, with RF and LR achieving moderate predictive performance for ATC. Peltan et al [27] predicted the occurrence of prehospital ATC in 285 severely injured patients, defining coagulopathy as an INR>1.5, with an AUROC value of only 0.73. Previous studies had small sample sizes and moderate predictive power, and were limited to single centers without external validation. In contrast, our multicenter study employed 10 ML models, with 3 demonstrating excellent performance. It included diverse medical centers and populations (White, Asian, Black, and Latino) and featured external validation, showcasing the models’ broad applicability and generalizability.

### Preoperative Risk Factors and In-Hospital Complications of PPTIC in Patients With Trauma

The definition of TIC relies primarily on coagulation tests, drawing from extensive literature on the subject. Brohi et al [3] recommended that patients with a PT > 18 s, an INR > 1.5, an APTT>60 s, or a TT>15 s could be diagnosed with the hypocoagulable phenotype of TIC, and this diagnosis was not affected by the use of anticoagulant medications or abnormal blood samples. MacLeod et al [28] predicted early coagulopathy in 20,103 patients with trauma and reported a higher incidence after trauma, which was associated with mortality. They defined early coagulopathy as a PT>14 s, an APTT>35 s, and a PLT<100×10^9/L, highlighting its role as an independent predictor of death despite other risk factors [28]. Currently, the accepted definition of TIC utilizes the INR, with an INR>1.2 being the threshold for detecting TIC [8]. Yuan et al [18] defined TIC for predicting mortality among patients with trauma as a PLT<100×10^9^/L, an INR>1.25, a PT>14 seconds, and an APTT>36 seconds [18]. We defined TIC based on coagulation test reference ranges from various centers and literature, using slightly lower standards than Brohi et al [3]. The main reasons for adopting these criteria are as follows: (1) lower diagnostic criteria alert clinicians to take early preventive measures to prevent PPTIC from progressing to disseminated intravascular coagulation; and (2) patients with preexisting TIC have a 50% chance of persisting with TIC postoperatively.

The prolongation of the preoperative APTT, PT, and INR in patients with trauma is understandable as a risk factor for PPTIC. The decreases in hemoglobin, hematocrit, and RBC levels and lower admission DBP are associated mainly with acute anemia due to preoperative bleeding in patients with trauma. Ryan et al [29] noted a close correlation between initial low hematocrit or hemoglobin levels in patients with trauma and hemorrhagic shock. RBC and hematocrit levels are key factors in platelet transport and adhesion to endothelial injury. Platelet adhesion increases 5-fold when hematocrit rises from 10% to 40% [30]. Therefore, the RBC level is closely related to the platelet level. Steele et al [31] reported an independent correlation between hyponatremia at admission and hypocalcemia. Ionic calcium is crucial not only for the formation and stability of fibrin polymerization sites but also for many platelet-related functions [32]. Platelet activation and aggregation are also calcium-dependent [33] and play crucial roles in the pathophysiology of TIC [34]. Elevated ALT and AST levels indicate liver damage, which impacts coagulation factor production and the coagulation process.

Hess et al [35] reported that patients with TIC experience more blood loss, higher transfusion needs, and a greater incidence of MODS compared to those without TIC. Brohi et al [3] highlighted that TIC is associated with a 4-fold increase in mortality, increased rates of massive transfusion and MODS, and prolonged ICU and LOS. Smalls et al [36] reported that patients with TIC due to splenic injury face 2.4 times more complications, with increased mortality (1.3-fold), sepsis (2-fold), acute respiratory distress syndrome (2.6-fold), acute renal failure (1.5-fold), and cardiac arrest (1.5-fold). These findings align with our study, where patients with PPTIC had increased risks of sepsis (1.75-fold), heart failure (1.5-fold), delirium (3.08-fold), abnormal coagulation (3.57-fold), tracheostomy (2.76-fold), mortality (2.19-fold), urinary tract infection (1.95-fold), and longer LOS and ICU stay.

### Limitations

This retrospective study focused primarily on predicting PPTIC in patients with trauma via admission laboratory indicators and medical records. In this study, we addressed missing data to varying extents by excluding variables with more than 40% missing values and applying distinct imputation methods for variables with less than 40% missing data (KNN for continuous variables and multiple imputation for categorical variables). While multiple imputation is an effective method to mitigate systematic bias from nonrandom missing data, some data remained missing, which could affect the model’s predictive accuracy for specific patient characteristics. Due to missing values exceeding 40% for certain indicators, such as height, admission peripheral capillary oxygen saturation, temperature on admission, pre-lymphocyte, pre-total protein, pre-globulin, pre-direct bilirubin, pre-lactate dehydrogenase, pre-D-dimer, and post-D-dimer (Multimedia Appendix 3), these variables were excluded from model development. As these missing data may impact the predictive performance of the model, future studies could address this by collecting more comprehensive data to improve model robustness. Additionally, large-sample prospective clinical trials are necessary to validate the model’s applicability in clinical settings and assess its effectiveness in reducing the incidence of PPTIC.

### Conclusions

Our study, using data from 4 medical centers, identified risk factors for PPTIC in patients with trauma. Significant factors included (1) prolonged preoperative APTT, PT, and INR; (2) decreased hemoglobin, hematocrit, RBC, calcium, and sodium levels; (3) lower admission DBP; (4) elevated ALT and AST levels; (5) increased admission HR; and (6) the need for emergency surgery or perioperative transfusion. PPTIC was associated with higher rates of sepsis, heart failure, delirium, abnormal coagulation, tracheostomy, mortality, and extended hospital and ICU stays. Of the 10 ML models evaluated for predicting PPTIC, RF demonstrated the best performance with an AUROC of 0.91, AUPRC of 0.89, accuracy of 0.84, and a BS of 0.13.

## Data Availability

The datasets generated during and/or analyzed during this study are not publicly available due to ongoing research and unpublished results but are available from the corresponding author on reasonable request.

## Appendix

Multimedia Appendix 1 The TRIPOD-AI (Transparent Reporting of a Multivariable Prediction Model for Individual Prognosis or Diagnosis—Artificial Intelligence) guidelines.

Multimedia Appendix 2 A comprehensive description of the methodology.

Multimedia Appendix 3 The amount of data missing and the coding process.

Multimedia Appendix 4 Sklearn packages for machine learning models.

Multimedia Appendix 5 Python packages used for data processing and analysis.

Multimedia Appendix 6 Ten machine learning models predicting patients with trauma who have preoperative and postoperative traumatic coagulopathy and related performance evaluation.

Multimedia Appendix 7 Results of 5-fold cross-validation for 10 machine learning models.

Multimedia Appendix 8 SHA*P* values of AdaBoost, DT, KNN, LR, NB, NN, and SVM for predicting the risk of TIC before and after surgery in patients with trauma. DT: decision tree; KNN: k-nearest neighbor; LR: logistic regression; NB: naive Bayes; NN: neural network; SVM: support vector machine; SHAP: Shapley additive explanation; TIC: traumatic coagulopathy.

## Abbreviations

AI: artificial intelligence
ALT: alanine aminotransferase
APTT: activated partial thromboplastin time
AST: aspartate amino transferase
AUPRC: area under the precision-recall curve
AUROC: area under the receiver operating characteristic curve
BS: Brier score
DBP: diastolic blood pressure
DT: decision tree
GB: gradient boosting
HR: heart rate
ICU: intensive care unit
INR: international normalized ratio
KNN: k-nearest neighbor
LOS: length of stay
LR: logistic regression
ML: machine learning
MODS: multiple organ dysfunction syndrome
MOF: multiple organ failure
NB: naive Bayes
NN: neural networks
PLT: platelet count
PPTIC: preoperative and postoperative traumatic coagulopathy
PT: prothrombin time
RBC: red blood cell
STROCSS: Strengthening the Reporting of Cohort Studies in Surgery
TRIPOD-AI: Transparent Reporting of a Multivariable Prediction Model for Individual Prognosis or Diagnosis—Artificial Intelligence
RF: random forest
SHAP: Shapley additive explanation
SVM: support vector machine
TIC: traumatic coagulopathy
XGBoost: extreme gradient boosting

## References

[1] GBD 2017 Causes of Death Collaborators (2018). Global, regional, and national age-sex-specific mortality for 282 causes of death in 195 countries and territories, 1980-2017: a systematic analysis for the Global Burden of Disease Study 2017. Lancet.

[2] Kornblith LZ, Moore HB, Cohen MJ (2019). Trauma-induced coagulopathy: the past, present, and future. J Thromb Haemost.

[3] Brohi K, Singh J, Heron M, Coats T (2003). Acute traumatic coagulopathy. J Trauma.

[4] Maegele M, Lefering R, Yucel N, Tjardes T, Rixen D, Paffrath T, Simanski C, Neugebauer E, Bouillon B (2007). Early coagulopathy in multiple injury: an analysis from the German Trauma Registry on 8724 patients. Injury.

[5] Harhangi BS, Kompanje EJO, Leebeek FWG, Maas AIR (2008). Coagulation disorders after traumatic brain injury. Acta Neurochir.

[6] Duque P, Calvo A, Lockie C, Schöchl H (2021). Pathophysiology of trauma-induced coagulopathy. Transfus Med Rev.

[7] Cohen MJ, Christie SA (2017). Coagulopathy of trauma. Crit Care Clin.

[8] Cole E, Weaver A, Gall L, West A, Nevin D, Tallach R, O'Neill B, Lahiri S, Allard S, Tai N, Davenport R, Green L, Brohi K (2021). A decade of damage control resuscitation: new transfusion practice, new survivors, new directions. Ann Surg.

[9] Rossaint R, Afshari A, Bouillon B, Cerny V, Cimpoesu D, Curry N, Duranteau J, Filipescu D, Grottke O, Grønlykke L, Harrois A, Hunt BJ, Kaserer A, Komadina R, Madsen MH, Maegele M, Mora L, Riddez L, Romero CS, Samama CM, Vincent JL, Wiberg S, Spahn DR (2023). The European guideline on management of major bleeding and coagulopathy following trauma: sixth edition. Crit Care.

[10] Whicher D, Ahmed M, Thadaney Israni S, Matheny M, National Academy of Medicine (2023). Artificial Intelligence in Health Care: The Hope, the Hype, the Promise, the Peril.

[11] Sidey-Gibbons JAM, Sidey-Gibbons CJ (2019). Machine learning in medicine: a practical introduction. BMC Med Res Methodol.

[12] Liu NT, Salinas J (2017). Machine learning for predicting outcomes in trauma. Shock.

[13] Seki T, Kawazoe Y, Ohe K (2021). Machine learning-based prediction of in-hospital mortality using admission laboratory data: a retrospective, single-site study using electronic health record data. PLoS One.

[14] Rashidi HH, Bowers KA, Reyes Gil M (2023). Machine learning in the coagulation and hemostasis arena: an overview and evaluation of methods, review of literature, and future directions. J Thromb Haemost.

[15] Johnson A, Bulgarelli L, Pollard T, Gow B, Moody B, Horng S, Anthony Celi L, Mark R (2021). MIMIC-IV. PhysioNet.

[16] Collins GS, Moons KGM, Dhiman P, Riley RD, Beam AL, van Calster B, Ghassemi M, Liu X, Reitsma JB, van Smeden M, Boulesteix AL, Camaradou JC, Celi LA, Denaxas S, Denniston AK, Glocker B, Golub RM, Harvey H, Heinze G, Hoffman MM, Kengne AP, Lam E, Lee N, Loder EW, Maier-Hein L, Mateen BA, McCradden MD, Oakden-Rayner L, Ordish J, Parnell R, Rose S, Singh K, Wynants L, Logullo P (2024). TRIPOD+AI statement: updated guidance for reporting clinical prediction models that use regression or machine learning methods. BMJ.

[17] Mathew G, Agha R, Albrecht J, Goel P, Mukherjee I, Pai P, D'Cruz AK, Nixon IJ, Roberto K, Enam SA, Basu S, Muensterer OJ, Giordano S, Pagano D, Machado-Aranda D, Bradley PJ, Bashashati M, Thoma A, Afifi RY, Johnston M, Challacombe B, Ngu JCY, Chalkoo M, Raveendran K, Hoffman JR, Kirshtein B, Lau WY, Thorat MA, Miguel D, Beamish AJ, Roy G, Healy D, Ather HM, Raja SG, Mei Z, Manning TG, Kasivisvanathan V, Rivas JG, Coppola R, Ekser B, Karanth VL, Kadioglu H, Valmasoni M, Noureldin A (2021). STROCSS 2021: strengthening the reporting of cohort, cross-sectional and case-control studies in surgery. Int J Surg.

[18] Yuan Q, Yu J, Wu X, Sun YR, Li ZQ, Du ZY, Wu XH, Hu J (2018). Prognostic value of coagulation tests for in-hospital mortality in patients with traumatic brain injury. Scand J Trauma Resusc Emerg Med.

[19] PPTIC-ML. GitHub.

[20] Yu M, Wang S, He K, Teng F, Deng J, Guo S, Yin X, Lu Q, Gu W (2024). Predicting the complexity and mortality of polytrauma patients with machine learning models. Sci Rep.

[21] Wang K, Tian J, Zheng C, Yang H, Ren J, Liu Y, Han Q, Zhang Y (2021). Interpretable prediction of 3-year all-cause mortality in patients with heart failure caused by coronary heart disease based on machine learning and SHAP. Comput Biol Med.

[22] Uddin S, Khan A, Hossain ME, Moni MA (2019). Comparing different supervised machine learning algorithms for disease prediction. BMC Med Inform Decis Mak.

[23] Badillo S, Banfai B, Birzele F, Davydov II, Hutchinson L, Kam-Thong T, Siebourg-Polster J, Steiert B, Zhang JD (2020). An introduction to machine learning. Clin Pharmacol Ther.

[24] Alderden J, Pepper GA, Wilson A, Whitney JD, Richardson S, Butcher R, Jo Y, Cummins MR (2018). Predicting pressure injury in critical care patients: a machine-learning model. Am J Crit Care.

[25] Perkins ZB, Yet B, Marsden M, Glasgow S, Marsh W, Davenport R, Brohi K, Tai NRM (2021). Early identification of trauma-induced coagulopathy: development and validation of a multivariable risk prediction model. Ann Surg.

[26] Li K, Wu H, Pan F, Chen L, Feng C, Liu Y, Hui H, Cai X, Che H, Ma Y, Li T (2020). A machine learning-based model to predict acute traumatic coagulopathy in trauma patients upon emergency hospitalization. Clin Appl Thromb Hemost.

[27] Peltan ID, Rowhani-Rahbar A, Vande Vusse LK, Caldwell E, Rea TD, Maier RV, Watkins TR (2016). Development and validation of a prehospital prediction model for acute traumatic coagulopathy. Crit Care.

[28] MacLeod JBA, Lynn M, McKenney MG, Cohn SM, Murtha M (2003). Early coagulopathy predicts mortality in trauma. J Trauma.

[29] Ryan ML, Thorson CM, Otero CA, Vu T, Schulman CI, Livingstone AS, Proctor KG (2012). Initial hematocrit in trauma: A paradigm shift?. J Trauma Acute Care Surg.

[30] Turitto VT, Weiss HJ (1980). Red blood cells: their dual role in thrombus formation. Science.

[31] Steele T, Kolamunnage-Dona R, Downey C, Toh C, Welters I (2013). Assessment and clinical course of hypocalcemia in critical illness. Crit Care.

[32] Lier H, Krep H, Schroeder S, Stuber F (2008). Preconditions of hemostasis in trauma: a review. The influence of acidosis, hypocalcemia, anemia, and hypothermia on functional hemostasis in trauma. J Trauma.

[33] Matthay ZA, Fields AT, Nunez-Garcia B, Patel MH, Cohen MJ, Callcut RA, Kornblith LZ (2020). Dynamic effects of calcium on in vivo and ex vivo platelet behavior after trauma. J Trauma Acute Care Surg.

[34] Vulliamy P, Gillespie S, Armstrong PC, Allan HE, Warner TD, Brohi K (2019). Histone H4 induces platelet ballooning and microparticle release during trauma hemorrhage. Proc Natl Acad Sci U S A.

[35] Hess JR, Brohi K, Dutton RP, Hauser CJ, Holcomb JB, Kluger Y, Mackway-Jones K, Parr MJ, Rizoli SB, Yukioka T, Hoyt DB, Bouillon B (2008). The coagulopathy of trauma: a review of mechanisms. J Trauma.

[36] Smalls N, Obirieze A, Ehanire I (2015). The impact of coagulopathy on traumatic splenic injuries. Am J Surg.

