# Supplementary material for: Ten Machine Learning Models for Predicting Preoperative and Postoperative Coagulopathy in Patients With Trauma: Multicenter Cohort Study
Source: J Med Internet Res. 2025 Jan 22;27:e66612. doi: 10.2196/66612 (PMC11799815; doi:10.2196/66612)
Supplement: Multimedia Appendix 2 [file jmir_v27i1e66612_app2.docx]

A comprehensive description of the methodology.

| **1. Keywords Extracted**  To identify trauma patients from the MIMIC-IV database, we utilized specific keywords related to ICU events and procedures:   - **ICU keywords**:   traffic  knife  fall   - **Procedure keywords**:   fracture  Intracranial hematoma  Skull injury  Keywords describing "suture of laceration" for various organs, such as:   - - - Closure of laceration of liver     - Suture of laceration of bladder     - Hemopneumothorax     - Hemothorax   These procedure-related keywords were chosen to identify specific surgeries or treatments commonly associated with trauma cases.  **2. Method of extracting keywords**  The extraction process was performed using a structured SQL query with the following key steps:   - **Pattern matching with ILIKE**:   We employed the ILIKE '%keyword%' clause, a case-insensitive pattern-matching |
| --- |
| operator in SQL, to locate records that contain partial matches to these keywords, irrespective of capitalization.   - **Keyword extraction**:   ICU-related and procedure-related keywords were processed separately using two distinct UNNEST statements:   - - - **ICU keywords**: A temporary table (icuk) containing ICU-related keywords was created and used to match the long_title field in the mimiciv_hosp.d_icd_diagnoses table, identifying relevant records that also exist in the mimiciv_icu.icustays table.     - **Procedure keywords**: A temporary table (pk) was created with procedure-related keywords and matched against the long_title field in the procedures_icd table to identify trauma-related procedures. - **Keyword matching**:   Each keyword array was left-joined with the corresponding diagnosis or procedure descriptions to ensure all relevant entries were included.  **3. Storing the extracted results**   - The matched ICU and procedure keywords were aggregated using the string_agg function to form two distinct columns: icu_keyword and procedure_keyword, with entries separated by the special character ‘￥’ for clarity.   Additional information, including diagnosis descriptions, start and end times, and other relevant data, was aggregated and calculated to provide a comprehensive dataset for downstream analysis. |
