# Supplementary material for: Ten Machine Learning Models for Predicting Preoperative and Postoperative Coagulopathy in Patients With Trauma: Multicenter Cohort Study
Source: J Med Internet Res. 2025 Jan 22;27:e66612. doi: 10.2196/66612 (PMC11799815; doi:10.2196/66612)
Supplement: Multimedia Appendix 3 [file jmir_v27i1e66612_app3.docx]

**Table S1.** The amount of data missing and the coding process.

| Variables | Data types | Coding processing | Data volume | Data missing rate |
| --- | --- | --- | --- | --- |
| PE | Binary | One-Hot Encoding | 13236 | 0% |
| Sepsis | Binary | One-Hot Encoding | 10023 | 24% |
| Septicemia | Binary | One-Hot Encoding | 10023 | 24% |
| Perioperative blood transfusion | Binary | One-Hot Encoding | 13236 | 0% |
| Heart failure | Binary | One-Hot Encoding | 10023 | 24% |
| Delirium | Binary | One-Hot Encoding | 10023 | 24% |
| Hemothorax | Binary | One-Hot Encoding | 13236 | 0% |
| Hemopneumothorax | Binary | One-Hot Encoding | 13236 | 0% |
| Pulmonary edema | Binary | One-Hot Encoding | 10023 | 24% |
| Abnormal coagulation | Binary | One-Hot Encoding | 10023 | 24% |
| Endotracheal tube | Binary | One-Hot Encoding | 10023 | 24% |
| Invasive mechanical ventilation | Binary | One-Hot Encoding | 10023 | 24% |
| Trach | Binary | One-Hot Encoding | 10023 | 24% |
| Hypercholesterolemia | Binary | One-Hot Encoding | 10023 | 24% |
| End stage renal disease | Binary | One-Hot Encoding | 10023 | 24% |
| Systemic inflammatory response | Binary | One-Hot Encoding | 10023 | 24% |
| Acute respiratory distress syndrome | Binary | One-Hot Encoding | 10023 | 24% |
| Shock | Binary | One-Hot Encoding | 13236 | 0% |
| COPD | Binary | One-Hot Encoding | 13236 | 0% |
| Pleural effusion | Binary | One-Hot Encoding | 13236 | 0% |
| Cerebrovascular disease | Binary | One-Hot Encoding | 13236 | 0% |
| Cardiac arrest | Binary | One-Hot Encoding | 10023 | 24% |
| Anemia | Binary | One-Hot Encoding | 13236 | 0% |
| Post-VTE | Binary | One-Hot Encoding | 13236 | 0% |
| Diabetes | Binary | One-Hot Encoding | 13236 | 0% |
| HBP | Binary | One-Hot Encoding | 13236 | 0% |
| Pulmonary edema | Binary | One-Hot Encoding | 10023 | 24% |
| Renal failure | Binary | One-Hot Encoding | 10023 | 24% |
| Acute renal failure | Binary | One-Hot Encoding | 10023 | 24% |
| CHD | Binary | One-Hot Encoding | 13236 | 0% |
| Urinary tract infection | Binary | One-Hot Encoding | 10023 | 24% |
| Respiratory failure | Binary | One-Hot Encoding | 13236 | 0% |
| Pneumonia | Binary | One-Hot Encoding | 13236 | 0% |
| LOS | Continuous |  | 13236 | 0% |
| In-hospital death | Continuous |  | 13236 | 0% |
| LOS of ICU | Continuous |  | 5080 | 62% |
| Sex | Binary | One-Hot Encoding | 13236 | 0% |
| Age | Continuous |  | 13236 | 0% |
| Height | Continuous |  | 6701 | 49% |
| Weight | Continuous |  | 10183 | 23% |
| BMI | Continuous |  | 7413 | 44% |
| Emergency | Binary | One-Hot Encoding | 13236 | 0% |
| Admission HR | Continuous |  | 9555 | 28% |
| Admission SPO2 | Continuous |  | 6275 | 53% |
| Admission Temperature | Continuous |  | 6183 | 53% |
| Admission SBP | Continuous |  | 10831 | 18% |
| Admission DBP | Continuous |  | 10830 | 18% |
| Race | Multiple | One-Hot Encoding | 13236 | 0% |
| Pre-hematocrit | Continuous |  | 12963 | 2% |
| Pre-hemoglobin | Continuous |  | 13035 | 2% |
| Pre-RDW | Continuous |  | 12657 | 4% |
| Pre-RBC | Continuous |  | 12996 | 2% |
| Pre-WBC | Continuous |  | 12635 | 5% |
| Pre-lymphocyte % | Continuous |  | 8293 | 37% |
| Pre-monocyte % | Continuous |  | 8321 | 37% |
| Pre-eosinophil % | Continuous |  | 8333 | 37% |
| Pre-basophil % | Continuous |  | 8333 | 37% |
| Pre-MCH | Continuous |  | 12995 | 2% |
| Pre-MCHC | Continuous |  | 12995 | 2% |
| Pre-MCV | Continuous |  | 9705 | 27% |
| Pre-basophil | Continuous |  | 5065 | 62% |
| Pre-eosinophil | Continuous |  | 5065 | 62% |
| Pre-lymphocyte | Continuous |  | 5093 | 62% |
| Pre-monocyte | Continuous |  | 5064 | 62% |
| Pre-neutrophil | Continuous |  | 5065 | 62% |
| Pre-ALT | Continuous |  | 8382 | 37% |
| Pre-AST | Continuous |  | 8368 | 37% |
| Pre-GGT | Continuous |  | 3211 | 76% |
| Pre-TP | Continuous |  | 3476 | 74% |
| Pre-GLB | Continuous |  | 3270 | 75% |
| Pre-albumin | Continuous |  | 7457 | 44% |
| Pre-ALP | Continuous |  | 8455 | 36% |
| Pre-DBIL | Continuous |  | 3544 | 73% |
| Pre-IBIL | Continuous |  | 3592 | 73% |
| Pre-TBIL | Continuous |  | 8435 | 36% |
| Pre-LDH | Continuous |  | 5347 | 60% |
| Pre-chloride | Continuous |  | 10039 | 24% |
| Pre-calcium | Continuous |  | 11249 | 15% |
| Pre-creatinine | Continuous |  | 12465 | 6% |
| Pre-potassium | Continuous |  | 11936 | 10% |
| Pre-sodium | Continuous |  | 11791 | 11% |
| Pre-magnesium | Continuous |  | 8268 | 38% |
| Pre-AG | Continuous |  | 8246 | 38% |
| Pre-uric | Continuous |  | 3537 | 73% |
| Pre-glucose | Continuous |  | 2549 | 81% |
| Pre-PT | Continuous |  | 12767 | 4% |
| Pre-APTT | Continuous |  | 13236 | 0% |
| Pre-PLT | Continuous |  | 12586 | 5% |
| Pre-D-dimer | Continuous |  | 2845 | 79% |
| Pre-INR | Continuous |  | 13081 | 1% |
| Post-INR | Continuous |  | 13115 | 1% |
| Post-PT | Continuous |  | 12904 | 3% |
| Post-APTT | Continuous |  | 13236 | 0% |
| Post-PLT | Continuous |  | 12856 | 3% |
| Post-D-dimer | Continuous |  | 3196 | 76% |

APTT: activated partial thromboplastin time; ALT: alanine aminotransferase; ALP: alkaline phosphatase; AST: aspartate amino transferase; BMI: body mass index; BS: Brier score; COPD: Chronic obstructive pulmonary disease; Crea: creatinine; DBP: Diastolic blood pressure; DIBL: direct bilirubin; GGT: Glutamyl transferase; GB: Gradient boosting; HR: heart rate; HBP: high blood pressure; IBIL: indirect bilirubin; ICU: intensive care unit; INR: international normalized ratio; LDH: lactate dehydrogenase; LOS: length of stay; MCHC: Mean corpuscular hemoglobin concentration; MCV: mean corpuscular volume; MPV: mean platelet volume; PLT: platelet count; PDW: platelet distribution width; PT: prothrombin time; PE: Pulmonary embolism; RBC: Red blood cell; RDW: red cell distribution width; SBP: systolic pressure; TBIL: total bilirubin; TP: Total protein; WBC: white blood cell count
