# Supplementary material for: Ten Machine Learning Models for Predicting Preoperative and Postoperative Coagulopathy in Patients With Trauma: Multicenter Cohort Study
Source: J Med Internet Res. 2025 Jan 22;27:e66612. doi: 10.2196/66612 (PMC11799815; doi:10.2196/66612)
Supplement: Multimedia Appendix 4 [file jmir_v27i1e66612_app4.docx]

**Multimedia Appendix 4.** Sklearn packages for machine learning models.

| Machine learning models | The sklearn package |
| --- | --- |
| Logistic regression | sklearn.linear_model import LogisticRegression |
| Random forest | sklearn.ensemble import RandomForest Classifier |
| Support vector machine | sklearn.svm import SVC |
| Decision tree | sklearn.tree import DecisionTreeClassifier |
| K-Nearest neighbors | sklearn.neighbors import KNeighborsClassifier |
| Gradient boosting | sklearn.ensemble import GradientBoostingClassifier |
| Neural networks | sklearn.neural_network import MLPClassifier |
| Naive bayes | sklearn.naive_bayes import GaussianNB |
| AdaBoost | sklearn.ensemble import AdaBoostClassifier |
| XGBoost | sklearn.xgboost import XGBClassifier |
