# Supplementary material for: Ten Machine Learning Models for Predicting Preoperative and Postoperative Coagulopathy in Patients With Trauma: Multicenter Cohort Study
Source: J Med Internet Res. 2025 Jan 22;27:e66612. doi: 10.2196/66612 (PMC11799815; doi:10.2196/66612)
Supplement: Multimedia Appendix 5 [file jmir_v27i1e66612_app5.docx]

**Multimedia Appendix 5.** Python packages used for data processing and analysis.

| **Step** | **Python Package/Library Used** |
| --- | --- |
| Data reading and loading | pandas |
| Data cleaning and missing value handling | pandas, numpy, KNNImputer (sklearn.impute) |
| Feature engineering | numpy, pandas, np.where (numpy), sklearn (sklearn.feature_selection, sklearn.linear_model) |
| Data normalization or standardization | StandardScaler (sklearn.preprocessing) |
| Data balancing | SMOTE (imblearn.over_sampling), RandomUnderSampler (imblearn.under_sampling) |
| Data splitting | train_test_split (sklearn.model_selection) |
| Model training | sklearn (e.g., LogisticRegression, RandomForestClassifier, SVC, GradientBoostingClassifier, etc.) |
| Hyperparameter tuning | GridSearchCV (sklearn.model_selection) |
| Model performance evaluation | roc_curve, auc, accuracy_score, precision_score, recall_score, f1_score, brier_score_loss (sklearn.metrics) |
| Model calibration | calibration_curve (sklearn.calibration) |
| Model explainability | shap |
| Data visualization | matplotlib, shap |
