# Supplementary material for: Ten Machine Learning Models for Predicting Preoperative and Postoperative Coagulopathy in Patients With Trauma: Multicenter Cohort Study
Source: J Med Internet Res. 2025 Jan 22;27:e66612. doi: 10.2196/66612 (PMC11799815; doi:10.2196/66612)
Supplement: Multimedia Appendix 7 [file jmir_v27i1e66612_app7.docx]

**Multimedia Appendix 7.** Results of five-fold cross-validation for 10 machine learning models.

|  | AUROC | Precision | Accuracy | Recall | F1 | Logloss | Meansquared error |
| --- | --- | --- | --- | --- | --- | --- | --- |
| LR1 | 0.89 | 0.84 | 0.84 | 0.84 | 0.84 | -0.42 | -0.16 |
| LR2 | 0.92 | 0.86 | 0.86 | 0.86 | 0.86 | -0.37 | -0.14 |
| LR3 | 0.91 | 0.86 | 0.86 | 0.86 | 0.86 | -0.37 | -0.14 |
| LR4 | 0.92 | 0.85 | 0.86 | 0.86 | 0.85 | -0.36 | -0.14 |
| LR5 | 0.9 | 0.84 | 0.84 | 0.84 | 0.84 | -0.4 | -0.16 |
| RF1 | 0.92 | 0.88 | 0.88 | 0.88 | 0.88 | -0.34 | -0.12 |
| RF2 | 0.92 | 0.88 | 0.88 | 0.88 | 0.88 | -0.33 | -0.12 |
| RF3 | 0.94 | 0.91 | 0.91 | 0.91 | 0.91 | -0.31 | -0.09 |
| RF4 | 0.94 | 0.91 | 0.9 | 0.9 | 0.9 | -0.31 | -0.1 |
| RF5 | 0.94 | 0.9 | 0.9 | 0.9 | 0.9 | -0.31 | -0.1 |
| SVM1 | 0.9 | 0.85 | 0.85 | 0.85 | 0.85 | -0.39 | -0.15 |
| SVM2 | 0.92 | 0.87 | 0.87 | 0.87 | 0.87 | -0.37 | -0.13 |
| SVM3 | 0.91 | 0.85 | 0.85 | 0.85 | 0.85 | -0.38 | -0.15 |
| SVM4 | 0.92 | 0.88 | 0.88 | 0.88 | 0.88 | -0.35 | -0.12 |
| SVM5 | 0.91 | 0.84 | 0.84 | 0.84 | 0.84 | -0.38 | -0.16 |
| DT1 | 0.8 | 0.81 | 0.81 | 0.81 | 0.81 | -6.97 | -0.19 |
| DT2 | 0.81 | 0.81 | 0.81 | 0.81 | 0.81 | -6.81 | -0.19 |
| DT3 | 0.81 | 0.82 | 0.82 | 0.82 | 0.82 | -6.57 | -0.18 |
| DT4 | 0.81 | 0.81 | 0.8 | 0.8 | 0.8 | -7.06 | -0.2 |
| DT5 | 0.82 | 0.83 | 0.83 | 0.83 | 0.83 | -6.18 | -0.17 |
| KNN1 | 0.84 | 0.77 | 0.77 | 0.77 | 0.77 | -0.99 | -0.23 |
| KNN2 | 0.82 | 0.74 | 0.74 | 0.74 | 0.73 | -1.23 | -0.26 |
| KNN3 | 0.8 | 0.73 | 0.73 | 0.73 | 0.73 | -1.55 | -0.27 |
| KNN4 | 0.82 | 0.77 | 0.77 | 0.77 | 0.77 | -1.3 | -0.23 |
| KNN5 | 0.81 | 0.77 | 0.77 | 0.77 | 0.77 | -2.06 | -0.23 |
| GB1 | 0.92 | 0.88 | 0.88 | 0.88 | 0.88 | -0.34 | -0.12 |
| GB2 | 0.94 | 0.88 | 0.88 | 0.88 | 0.88 | -0.3 | -0.12 |
| GB3 | 0.94 | 0.91 | 0.91 | 0.91 | 0.91 | -0.29 | -0.09 |
| GB4 | 0.94 | 0.91 | 0.9 | 0.9 | 0.9 | -0.28 | -0.1 |
| GB5 | 0.93 | 0.89 | 0.89 | 0.89 | 0.89 | -0.31 | -0.11 |
| NN1 | 0.89 | 0.84 | 0.84 | 0.84 | 0.84 | -0.48 | -0.16 |
| NN2 | 0.92 | 0.85 | 0.85 | 0.85 | 0.85 | -0.38 | -0.15 |
| NN3 | 0.92 | 0.84 | 0.84 | 0.84 | 0.84 | -0.39 | -0.16 |
| NN4 | 0.93 | 0.86 | 0.86 | 0.86 | 0.86 | -0.35 | -0.14 |
| NN5 | 0.9 | 0.83 | 0.83 | 0.83 | 0.83 | -0.42 | -0.17 |
| NB1 | 0.85 | 0.75 | 0.76 | 0.76 | 0.75 | -0.89 | -0.24 |
| NB2 | 0.82 | 0.74 | 0.74 | 0.74 | 0.74 | -1.1 | -0.26 |
| NB3 | 0.81 | 0.72 | 0.72 | 0.72 | 0.72 | -1.07 | -0.28 |
| NB4 | 0.82 | 0.75 | 0.75 | 0.75 | 0.75 | -1.07 | -0.25 |
| NB5 | 0.86 | 0.77 | 0.77 | 0.77 | 0.77 | -0.8 | -0.23 |
| AdaBoost1 | 0.91 | 0.88 | 0.88 | 0.88 | 0.88 | -0.66 | -0.12 |
| AdaBoost2 | 0.93 | 0.89 | 0.89 | 0.89 | 0.89 | -0.67 | -0.11 |
| AdaBoost3 | 0.93 | 0.89 | 0.88 | 0.88 | 0.88 | -0.67 | -0.12 |
| AdaBoost4 | 0.93 | 0.88 | 0.88 | 0.88 | 0.88 | -0.67 | -0.12 |
| AdaBoost5 | 0.92 | 0.87 | 0.87 | 0.87 | 0.87 | -0.67 | -0.13 |
| XGBoost1 | 0.92 | 0.88 | 0.88 | 0.88 | 0.88 | -0.52 | -0.12 |
| XGBoost2 | 0.93 | 0.88 | 0.88 | 0.88 | 0.88 | -0.44 | -0.12 |
| XGBoost3 | 0.93 | 0.9 | 0.9 | 0.9 | 0.9 | -0.39 | -0.1 |
| XGBoost4 | 0.94 | 0.91 | 0.9 | 0.9 | 0.9 | -0.37 | -0.1 |
| XGBoost5 | 0.93 | 0.89 | 0.89 | 0.89 | 0.89 | -0.41 | -0.11 |

LR: Logistic regression; RF: Random forest; SVM: Support vector machine; DT: Decision tree; KNN: K-Nearest neighbors; GB: Gradient boosting; NN: Neural networks; NB: Naive bayes; AUROC: area under the receiver operating characteristic curve; F1: F-score
