# Supplementary material for: Ten Machine Learning Models for Predicting Preoperative and Postoperative Coagulopathy in Patients With Trauma: Multicenter Cohort Study
Source: J Med Internet Res. 2025 Jan 22;27:e66612. doi: 10.2196/66612 (PMC11799815; doi:10.2196/66612)
Supplement: Multimedia Appendix 8 [file jmir_v27i1e66612_app8.docx]

**Figure S1.** Shap values of AdaBoost, DT, KNN, LR, NB, NN and SVM for predicting the risk of TIC before and after surgery in patients with trauma.

APTT: activated partial thromboplastin time; PT: prothrombin time; RBC: Red Blood Cell; SBP: systolic pressure; HR: heart rate; ALT: alanine aminotransferase; AST: aspartate amino transferase; DBP: Diastolic blood pressure; RF: Random forest; GB: Gradient boosting; SHAP: Shapley additive explanations; TIC: traumatic coagulopathy.
